# A South American sebecid from the Miocene of Hispaniola documents the presence of apex predators in early West Indies ecosystems

**DOI:** 10.1098/rspb.2024.2891

**Published:** 2025-04-30

**Authors:** Lázaro W. Viñola López, Jorge Velez-Juarbe, Philippe Münch, Juan N. Almonte Milan, Pierre-Olivier Antoine, Laurent Marivaux, Osvaldo Jimenez-Vasquez, Jonathan Bloch

**Affiliations:** ^1^ Florida Museum of Natural History, University of Florida, Gainesville, FL, USA; ^2^ Negaunee Integrative Research Center, Field Museum of Natural History, Chicago, IL, USA; ^3^ Department of Mammalogy, Natural History Museum of Los Angeles County, Los Angeles, CA, USA; ^4^ Department of Sedimentology, Géosciences Montpellier, Montpellier, Occitanie, France; ^5^ Department of Paleobiology, Museo Nacional de Historia Natural ‘Prof. Eugenio de Jesús Marcano’, Santo Domingo, Dominican Republic; ^6^ Department of Paleontology, Institut des Sciences de l'Evolution de Montpellier (ISE-M), UMR 5554 CNRS/UM/IRD/EPHE, Montpellier, Hérault, France; ^7^ Department of Paleontology (CNRS), Institut des sciences de l'evolution, Montpellier, Hérault, France; ^8^ Gabinete de Arqueología de la Oficina del Historiador de La Habana, Havana, La Habana, Cuba

**Keywords:** Caribbean, extinction, *Sebecus*, palaeobiogeography, biodiversity museum

## Abstract

The absence of terrestrial apex predators on oceanic islands led to the evolution of endemic secondary apex predators like birds, snakes and crocodiles, and loss of defence mechanisms among species. These patterns are well documented in modern and Quaternary terrestrial communities of the West Indies, suggesting that biodiversity there assembled similarly through overwater dispersal. Here, we describe fossils of a terrestrial apex predator, a sebecid crocodyliform with South American origins from the late Neogene of Hispaniola that challenge this scenario. These fossils, along with other putative sebecid specimens from Cuba and Puerto Rico, show that deep-time Caribbean ecosystems more closely resembled coeval localities in South America than those of today. We argue that Plio-Pleistocene extinction of apex predators in the West Indies resulted in mesopredator release and other evolutionary patterns traditionally observed on oceanic islands. Adaptations to a terrestrial lifestyle documented for sebecids and the chronology of West Indian fossils strongly suggest that they reached the islands in the Eocene–Oligocene through transient land connections with South America or island hopping. Furthermore, sebecids persisted in the West Indies for at least five million years after their extinction in South America, preserving the last populations of notosuchians yet recovered from the fossil record.

## 1. Introduction

The West Indies had a high diversity of endemic terrestrial vertebrates during the late Quaternary, resulting from millions of years of dispersal events (largely from South America) and subsequent *in situ* evolutionary radiations across the Cenozoic. The mode and tempo of vertebrate colonizations have been debated for over a century [1,2]. Some studies argue that the Core Greater Antilles (CGA; Cuba–Hispaniola–Puerto Rico) were colonized through a short-lived land connection or string of islands known as GAARlandia around the Eocene/Oligocene transition (EOT) (e.g. [3–8]). Geological reconstructions of GAARlandia suggest that it coincided with a drop in eustatic sea level and the uplift of the Aves Ridge at the EOT, connecting part of the CGA with northern South America for nearly two million years [4]. Results from recent geotectonic studies in the Lesser Antilles suggest that large islands in this area were exposed several times between the Eocene and the Pliocene, and that the distance between Puerto Rico and northern South America was significantly shorter than previously estimated, potentially facilitating dispersals from the continent to the islands [9–11]. Meanwhile, other studies argue that the island communities, which lack carnivores, marsupials, ungulates, and several clades of frogs and reptiles, were assembled as a result of colonization through long-distance overwater dispersal [12–17]. The absence of specialized terrestrial carnivores on islands has long-lasting ecological effects that ripple down through terrestrial ecosystems, often associated with mesopredator release and secondary flightlessness among birds [18–20]. These effects are well documented in modern and late Quaternary communities in the Caribbean, where crocodiles and birds acted as top predators with secondary adaptations to a more terrestrial lifestyle [21–23], and numerous clades of birds evolved flightlessness and terrestrial specializations independently (e.g. [21,24–26]).

In the late Eocene to early Oligocene, the time interval proposed for the GAARlandia landspan, the terrestrial carnivore guild in South America included metatherian mammals (Sparassodonta), large snakes (Madtsoiidae), large birds (Phorusrhacidae) and notosuchian crocodyliforms (Sebecidae) [27,28]. Sebecids had distinctive labio-lingually compressed and serrated (ziphodont) teeth and postcranial adaptations to terrestriality. They inhabited Europe in the late Cretaceous and South America through most of the Cenozoic until the early Late Miocene [28–30]. Fossil ziphodont crocodyliform teeth have previously been recovered from the Early Miocene of Cuba [22], but due to the independent evolution of this dental morphology in notosuchians and at least two other neosuchian clades, taxonomic attribution to a specific group has been lacking [31]. Here, we report the first unambiguous record of a sebecid outside of South America during the Cenozoic and argue that these South American terrestrial apex predators were probably a dominant part of the food web in the West Indies for much of the Neogene.

## 2. Material and methods

### (a) Institutional abbreviations

FLMNH, Florida Museum of Natural History, Gainesville, FL, USA; LACM, Vertebrate Palaeontology Collection, Natural History Museum of Los Angeles County, Los Angeles, CA, USA; MNHNCu, Palaeontology collection, Museo Nacional de Historia Natural de Cuba, La Habana, Cuba; MNHNSD, Museo Nacional de Historia Natural ‘Prof. Eugenio de Jesús Marcano’, Santo Domingo, Dominican Republic; UM, Museum of Palaeontology, University of Michigan, Ann Arbor, IL, USA.

### (b) Specimens observed

*Boverisuchus vorax* (LACM 21260-64 56184-86; FMNH PR399, PR479; UM uncat.); *Sebecus icaeorhinus* (FMNH PR 861 cast of holotype); Sebecidae (FMNH PR 2828, PR 5109; LACM uncat.); *Borealosuchus wilsoni* (FMNH PR 1674); *Alligator mississippiensis* (FLMNH 39106); *Crocodylus rhombifer* (MNHNSD FOS 23.325); *Crocodylus acutus* (MNHNSD); Gavialidae (MNHNSD FOS 23.003; 23.005; 23.1029, 23.1030).

### (c) Micro-computed tomography scanning and imaging

We produced high-resolution X-ray computed tomography (micro-CT) scans for two vertebrae (MNHNSD FOS 23.1323, 23.1324) and three teeth (MNHNSD FOS 23.1325, MNHNCu P3035, P3115) using the Phoenix v|tome|x M dual tube system at the University of Florida’s Nanoscale Research Facility. Scanning was carried out using a 240 kV X-ray tube and a tungsten target at 100 kV and 200 mA with an exposure time of 200 ms, a composite average of three images per view and a 1 mm copper filter. The resulting X-ray data were converted into volumetric data using GE’s proprietary datos|x software and reconstructed in three dimensions using VG Studio Max 4.1.2023. High-resolution X-ray computed tomography (CT) scan for LACM 162454 was performed using a Bruker Skyscan 1273. Scanning was executed using a 130 kV source, an exposure time of 592 ms and a 1 mm aluminium filter. Digital images of the sebecid and planocraniid teeth were taken with Keyence VHX-7000 digital microscope using a Zs-20 lens.

## 3. Systematic palaeontology

Crocodylomorpha Walker, 1970; Crocodyliformes Hay, 1930; Notosuchia Gasparini, 1971 (*sensu* Ruiz *et al*., 2021); Sebecosuchia Simpson, 1937 (*sensu* Leardi *et al*., 2024); Sebecidae Simpson, 1937 (*sensu* Leardi *et al*., 2024); *Sebecus* Simpson, 1937.

cf. *Sebecus* sp.

### (a) Material

Third(?) cervical (MNHNSD FOS 23.1323) and anterior caudal (MNHNSD FOS 23.1324) vertebrae of adult specimens and an isolated tooth (MNHNSD FOS 23.1325).

### (b) Locality and age

The specimens described here were collected in the locality Paleo Pond 1, 57 km along the Juan Pablo II (RD7) highway on Sabana Grande de Boya, Dominican Republic (electronic supplementary material, figures S1 and S2). On this roadcut, sediments late Miocene–early Pliocene in age (7.14−4.57 Ma) of the Yanigua/Los Haitises Formation crop out [32] (see electronic supplementary material for detailed biostratigraphic context).

### (c) Description and comparison

**Figure 1.**
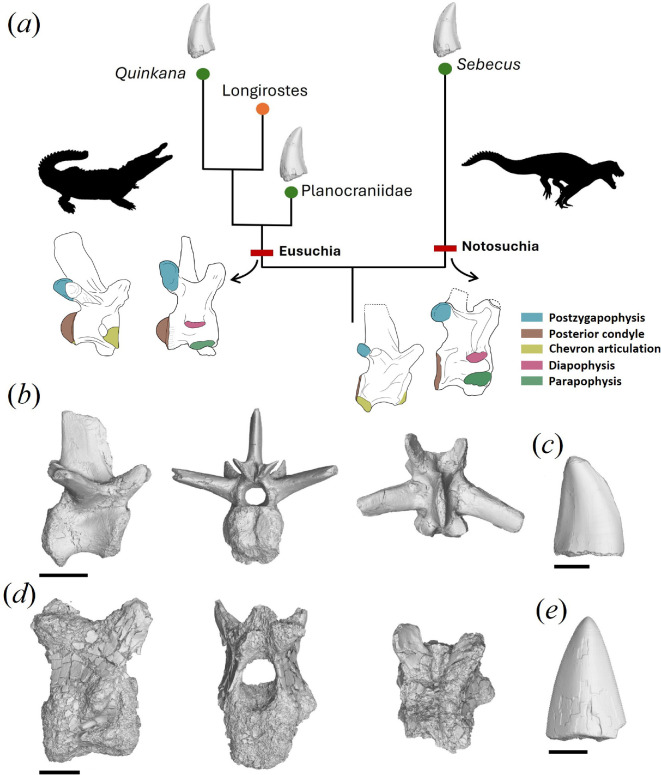
(*a*) Generalized relationship of crocodilians that independently evolved ziphodont dentition and the general anatomy of nuchal and caudal vertebrae in Notosuchia and Eusuchia. (*b*,*c*) Comparison of (*b*) caudal (MNHNSD FOS 23.1324) and (*c*) nuchal (MNHNSD FOS 23.1323) vertebrae of *Sebecus* sp. from the late Miocene–early Pliocene of Dominican Republic (scale bar = 15 mm). (*d*,*e*) Sebecid teeth from the (*d*) early Miocene of Cuba (MNHNCu P3115; scale bar = 10 mm) and (*e*) late Miocene–early Pliocene of Dominican Republic (MNHNSD FOS 23.1325; scale bar = 15 mm).

The cervical vertebra (MNHNSD FOS 23.1323) preserves most of its structures, but the surface cortical bone is highly fractured and eroded, obscuring some characters (figure 1*d*; electronic supplementary material, figure S3a). In some instances, the surface is completely eroded away, leaving a carbonate internal mould that outlines the general anatomy. The centrum is amphicoelous, anteroposteriorly longer than high or wide and the posterior articular surface area is larger than the anterior surface area. The centrum has a rectangular outline in lateral view, and the anterior and posterior articular surfaces are vertical and shallowly concave (nearly approaching an amphiplatyan condition) [33–38] with the anterior articular surface being higher than wide (anterior centrum height: 29 mm; anterior centrum width: 26 mm) (figure 1*d*; electronic supplementary material, figure S3a). The ventral surface of the centrum has a low median keel. The diapophysis is large, projects ventrolaterally and is located on the anterior half of the vertebra resembling the condition observed in the third cervical of *Sebecus icaeorhinus* and *Sahitisuchus fluminensis* [38,39], whereas in other basal mesoeucrocodylians (e.g. *Simosuchus clarki, Yacarerani boliviensis*), it is placed closer to the anterior margin [35,40]. The general outline of the parapophysis is preserved, from which it can be inferred that it was restricted to the anterior half of the vertebral centrum and that its main axis was oriented longitudinally and nearly horizontal, as in *S. icaeorhinus* and other mesoeucrocodylians (e.g. *S. clarki*, *Y. boliviensis*) (figure 1*d*; electronic supplementary material, figure S3a). The diapophysis and parapophysis are separated by a deep longitudinal groove on the lateral side of the centrum as in *S. icaeorhinus* [38]. The region where the hypapophysis would be located is eroded and missing in the specimens. The dorsal surface of the centrum has a relatively deep longitudinal groove along the midline. The neural canal is oriented anterodorsally to the horizontal plane. The anterior opening of the neural canal is nearly circular (17 mm wide, 12 mm high), contrasting with the tall and oval shape of the posterior opening (14 mm wide, 18 mm high) (electronic supplementary material, figure S3a).

Although most of the neural spine is missing, the base of the spine is posteriorly located, as in *Sebecus,* and unlike the centrally located spine of *Notosuchus* [36,37] (figure 1*d*; electronic supplementary material, figure S3a). The base of the spine is anteroposteriorly short [15] mm long; about one-third the length of the vertebra) as in *Sebecus*, *Notosuchus*, *Baurusuchus* and *Simosuchus* [35,38]. On the neural arch, anterior to the spine, there is a prespinal fossa resembling the condition in some mesoeucrocodylians (e.g. *Mahajangasuchus insignis*, *Baurusuchus albertoi*) and *Sebecus icaeorhinus*, although in MNHNSD FOS 23.1323, it is deeper and more developed as in the latter taxon [38,41,42] (S3a). This fossa is located on the midpoint between the spine and the prezygapophyses, and there is a longitudinal canal leading to it that starts at the anterodorsal margin of the arch between the prezygapophyses. The prezygapophyses are projected dorsally, as in most mesoeucrocodylians (e.g. *Mahajangasuchus*, *Simosuchus*, *Notosuchus*, *Baurusuchus*) and *Sebecus* [35,38,41,42]*,* and the anterior margin of the prezygapophyseal process has a small anteriorly oriented ridge. This anterior ridge resembles the prezygapophyseal bulge, resembling the condition observed in *Sebecus* and *Notosuchus,* but differs from other crocodilians with a convex process [38]. It further resembles *Sebecus* in that the articular facets of the prezygapophyses are flat, subcircular and oriented 45° with respect to the main axis of the vertebra (figure 1*d*; electronic supplementary material, figure S3a).

The anterior caudal vertebra (MNHNSD FOS 23.1324) is nearly complete and well preserved (figure 1*b*; electronic supplementary material, figure S3b). The neural spine is a long rectangular and medio-laterally compressed blade slightly inclined posteriorly, its base is anteroposteriorly broad (16.53 mm), and its width decreases apically. The neural spine is located on the postero-medial region of the neural arch, a condition shared with *Sebecus huilensis, Yacarerani boliviensis*, *Mahajangasuchus insignis, Campinasuchus dinizi* and baurusuchids [34,43]. The prezygapophyses are widely separated, projecting anterodorsally with the articular surface oriented dorsomedially. There is a deep prespinal fossa at the midline of the vertebra, between the prezygapophysis and anterior to the base of the neural spine. The post-zygapophyses are short, project ventrolaterally and are much more inclined than the prezygapophysis (electronic supplementary material, figure S3b). The transverse processes are oval in cross-section as in *Sebecus huilensis*, whereas they are smaller and dorsoventrally compressed in the advanced notosuchians *Marialiasuchus* and *Caipirasuchus* [34,44,45]. The transverse processes are located just ventral to the level of the pre- and post-zygapophyses, and project posterolaterally and slightly dorsal relative to the main axis of the vertebra (figure 1*b*; electronic supplementary material, figure S3b). On each side of the dorsal surface of the vertebrae, between the base of the transverse processes and the neural spine, there is a depression, which is also found in *Notosuchus terrestris* [37]. The neural canal is large, oval in cross-section anteriorly and nearly circular posteriorly (electronic supplementary material, figure S3b). The neural arch is fused to the centrum, thereby suggesting the specimen reached skeletal maturity (figure 1*b*).

In lateral view, the centrum is elongated and tall, and its lateral walls are excavated (figure 1*b*; electronic supplementary material, figure S3b). The centrum is amphicoelous and longer (24.14 mm) than wide (9.88 mm). In lateral view, the anterior articular surface has a circular outline and is anteroventrally inclined, whereas the posterior surface is subrectangular and slightly posterodorsally oriented. The ventral margin of the centrum forms an arch, with the posterior region of the centrum projecting more ventrally than the anterior one, resembling the prominent hemapophyses in the anterior caudals of *Simosuchus clarki*, *Mahajangasuchus insignis* and *Baurusuchus albertoi* [35,41,42]. The hemapophysis is inclined posteroventrally like in other sebecids, mesoeucrocodylians (*Mahajangasuchus*), sphagesaurians (e.g. *Mariliasuchus*) and baurusuchids (*Campinasuchus*) [30,34,41–44,46]. In ventral view, the midsection of the centrum is medio-laterally compressed, whereas the anterior and posterior surfaces expand laterally. The ventral surface of the centrum is excavated at the midline, forming two low parasagittal ridges.

MNHNSD FOS 23.1325 is the upper portion of a labio-lingually flattened tooth (electronic supplementary material, figure S4d). The preserved portion of the tooth is approximately 12 mm high; its base has a mesiodistal length of 8.11 mm and a labiolingual width of 3.55 mm (electronic supplementary material, figures S4d and S5b). As such, the length/width ratio of 2.3 is similar to that observed in *Sebecus* [47]. The lingual and labial surfaces are convex and smooth. In lingual and labial views, the tooth is nearly symmetrical along its longitudinal axis. The mesial and distal surfaces are narrow and bear a well-defined carinae with true serrations formed by clefts in the enamel (electronic supplementary material, figure S5b). The denticles are rectangular to convex, and the ones located in the middle portion of the teeth are approximately 0.2 mm in length, with a density of approximately 5 denticles per mm.

### (d) Other ziphodont teeth

Three other isolated teeth were collected from the early Miocene Domo de Zaza locality in Cuba (Museo Nacional de Historia Natural de Cuba, MNHNCu P3035, P3115) and the early Oligocene Yauco locality in Puerto Rico (Los Angeles County Museum, LACM 162454) (electronic supplementary material, figures S1, S4 and S5). The three teeth are labio-lingually compressed and slightly recurved mesially and lingually. The carinae are worn to different degrees but still show well-defined serrations formed of rectangular to convex denticles with a density of approximately 5 denticles per mm (see electronic supplementary material for detailed description of specimens and biostratigraphic data).

## 4. Discussion

### (a) Sebecids and ziphodont crocodyliforms in the West Indies

The oldest ziphodont crocodyliform recorded in the West Indies corresponds to an isolated tooth from the middle Eocene Seven Rivers locality in Jamaica [48]. During the middle Eocene, Western Jamaica was connected to the Nicaragua Rise, a southern extension of North America. Vertebrates thus far recovered from this locality have affinities with the fauna from similar age deposits in North America [49,50]. Given the biogeographical affinities of fossils from Jamaica, the ziphodont tooth from Seven Rivers most probably represents a planocraniid rather than a sebecid [31,51].

Other ziphodont crocodyliform teeth in the West Indies come from Domo de Zaza, a Lower Miocene (17.5−18.5 Ma) locality of the Lagunitas Formation in central Cuba [22,31]. Because at least three groups of Cenozoic crocodyliforms, including sebecosuchian, planocraniids and mekosuchines, independently evolved ziphodont dentition, assigning the Cuban isolated teeth to a specific group was initially problematic. Affinities with Mekosuchinae are unlikely because all members of this clade are part of a mostly endemic radiation from the Eocene-Pleistocene of Australia [52–54], and only a later Holocene dispersal into the Pacific Islands [55]. Meanwhile, a close relationship with planiocraniids was not entirely discarded as this clade inhabited North America and Eurasia, but the group became extinct in the Eocene [51]. Sebecosuchians are the other known clade in the Americas with ziphodont teeth, but their New World fossil record was, until now, restricted to South America in the Cenozoic [28,31]. Nonetheless, the amphicoelous vertebrae and the ziphodont tooth from the Late Miocene–Early Pliocene of the Dominican Republic show that sebecids inhabited the West Indies. Therefore, it is likely that the Early Miocene Cuban specimens as well as the early Oligocene ziphodont tooth from Puerto Rico also belong to insular sebecids (see electronic supplementary material for specimens description).

**Figure 2.**
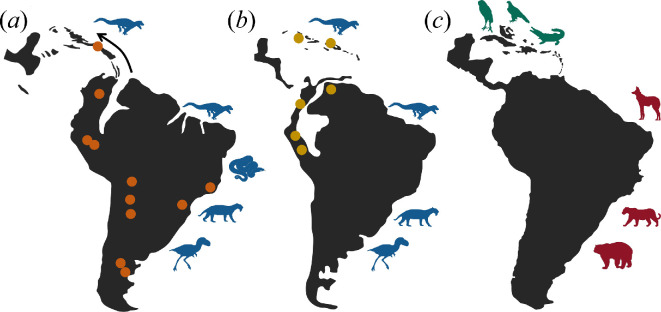
Maps of South America and the Caribbean region during the (*a*) Palaeogene, (*b*) Neogene and (*c*) late Quaternary show the generalized distribution of Sebecidae in circles. The silhouettes correspond to the terrestrial apex predator groups present in the region during each period, with native South American predators (Sebecidae, Madtsoiidae, Phorusrhacidae, Sparassodonta) in blue, late Cenozoic invasive predators (Canidae, Felidae, Ursidae) in red and endemic secondary terrestrial predators in the West Indies (Strigiformes, Accipitridae, Crocodylus) in green. Localities of Sebecidae based on electronic supplementary material, table S2.

In South America, the Palaeogene records of Sebecidae are best documented at high latitudes, but isolated specimens from Eocene deposits at low latitudes revealed that the family had a pancontinental distribution [28,46] (figure 2). Their range in the Miocene was restricted to the more tropical zones at mid and low latitudes, consistent with other groups of terrestrial vertebrates in South America [28,46]. How sebecids colonized the Greater Antilles is unclear, but the source population was probably in northern South America. Considering their terrestrial adaptations, their dispersal may have (i) been either facilitated by some ephemeral terrestrial connection or string of large and closely spaced islands or (ii) occurred on a natural raft. The fossil from the early Oligocene of Puerto Rico is consistent with the age and location where it would be expected to find a South American carnivore that dispersed through GAARlandia or the Lesser Antilles around the Eocene-Oligocene transition. Once in the CGA, the founding population would have become isolated as islands separated with the opening of the Mona passage in the Oligocene and the Mind passage in the Early Miocene [6,56]. This is consistent with their fossil record on the island, limited so far to the CGA. Similar temporal and spatial colonization patterns have been documented for other clades with South American affinities in the West Indies, like sloths, caviomorph rodents (chinchilloids) and anurans [7,8,57–59].

### (b) The ‘missing’ apex carnivores and their asynchronous extinction

Absence of native terrestrial carnivores in the late Quaternary fossil record and modern ecosystems in the West Indies has been cited as evidence against biogeographical hypotheses that propose the existence of a land connection with the mainland. This is not only reflected in the lack of taxa from the well-documented Quaternary fossil record in the region, but also in the endemic radiation of large nocturnal and diurnal raptorial birds that filled this ecological niche [21,26,60] along with *Crocodylus rhombifer,* one of the most terrestrially adapted crocodilians alive today [22,61]. These predators were at the top of the food chain in the CGA, which included more than 51 land mammal species (sloths, rodents, primates and island shrews), along with numerous other now extinct reptiles and birds. Fossils of these species are often found in predator roosts showing evidence of bitemarks and digestion [22,23,26]. Furthermore, several clades of birds, including rails, cave rails, cranes, ibis, as well as predatory owls, hawks and falcons, evolved adaptations to a terrestrial lifestyle and, in some cases, secondary flightlessness probably related to the lack of terrestrial predators [24,26]. These adaptations are probably fairly recent, following the disappearance of sebecids in the West Indies in post-Miocene times (figure 2). As it has been documented on other islands, extinction and occasional dispersal can change the structure and composition of an insular fauna originally assembled through vicariance into resembling a truly oceanic one [62].

In South America, these notosuchians were apex predators adapted to terrestrial ecosystems. The postcranial morphology of *Sebecus icaeorhinus* shows several adaptations to terrestriality with erect limb posture [36], while the neuroanatomy and cranial morphology of several species further support this habitat preference and suggest that prey acquisition and manipulation probably occurred on land [63–65]. Results from multiproxy isotope analyses (δ^13^C, δ^18^O, δ^43/42^Ca) of tooth enamel derived from Palaeocene fossils from Bolivia suggest that sebecids occupied a top position on the C3-based food web in a local dry tropical environment [66]. Sebecids in the West Indies were probably also apex predators in terrestrial ecosystems for more than 22 million years. Their remains have been found in association with fossils of sloths and other terrestrial (e.g. rodents, primates) or semiaquatic vertebrates (e.g. pelomedusoid turtles; electronic electronic supplementary material, table S1).

The last unequivocal records of Sebecidae from South America are from the early Late Miocene, including *Sebecus huilensis* (Peru and Colombia), *Barinasuchus arveloi* (Venezuela and Peru) and an undescribed large form known from the Villavieja Formation (12.58−10.52 Ma) in La Venta, Colombia [28,67–71]. The geographic range construction and subsequent extinction of sebecids has been associated with the progressive retreat of tropical climate to lower latitudes in South America through the Cenozoic, and habitat changes in the earliest Late Miocene caused by the disintegration of the Pebas wetland system [28,46]. In the West Indies, the last occurrence of sebecids dates from the late Miocene–early Pliocene (7.14−4.57 Ma) [32], at least 5 Myr later than on the mainland [28,70]. The Hispaniolan fossils described here document not only the last known sebecid, but also the last appearance of the clade Notosuchia. This highly diverse group of crocodyliforms with a wide array of ecological and morphological specialization had a nearly global distribution in the Cretaceous, of which only Sebecosuchia survived the Cretaceous/Palaeogene boundary in Europe and South America [72,73].

Islands (physical and ecological) around the world often act as biodiversity museums, hosting relics of old lineages that once had a broad distribution but that today are threatened by extinction [74,75]. These lineages survived and, in some instances, diversified in isolation on the islands for millions of years while the remaining populations on the mainland disappeared [74]. The West Indies are well known as the last refugium of numerous lineages of plants, invertebrates and vertebrates threatened by extinction or extinct elsewhere (e.g. [76–79]). The isolation of sebecids on the islands buffered them from macroecological changes recorded in South America, allowing them to survive longer than their relatives on the mainland. Moreover, the fauna associated with these last sebecids included other crocodyliforms (alligatoroids and gryposuchine gavialoids), pelomedusoid turtles, caviomorph rodents and megalocnid sloths [32] (electronic supplementary material, table S1), suggesting that the Cenozoic Greater Antillean vertebrate fauna more closely resembled that of the South American mainland than previously recognized, with multiple lineages of crocodyliforms as macropredators [80]. The relatively recent decline and demise of these multi-species crocodyliform assemblages probably paved the way for the origination of late Quaternary and modern-day trophic structures in the West Indies.

## Data Availability

All the data used in this study is available on the manuscript and the electronic supplementary material [81].
